# **Creation of a murine orthotopic hepatoma model with intra-abdominal metastasis**

**Published:** 2016

**Authors:** Jamie Harris, Andre Kajdacsy-Balla, Bill Chiu

**Affiliations:** 1 *Division of General Surgery, Rush University Medical Center. 1653 W. Congress Parkway Jelke Suite 792, Chicago, USA *; 2 *Division of Pathology, University of Illinois Chicago, 840 S. Wood St. Suite 130 CSN, Department of Pathology, Chicago, USA*; 3 *Division of Pediatric Surgery, University of Illinois Chicago, 840 S. Wood Street, Department of Surgery (MC 958) Room 416, Clinical Sciences Building, Chicago, USA *

**Keywords:** Hepatoma, Ultrasound, Metastatic, Mouse, Orthotopic

## Abstract

**Aim:**

To create an orthotopic hepatoma model with local metastasis monitored with ultrasound could be created as a platform for testing new treatments.

**Background:**

Hepatoma accounts for 25% of liver tumors in children with poor overall survival. Intraabdominal metastasis are present in 35% of patients at time of diagnosis. We hypothesized that an orthotopic tumor model with local metastasis could be created as a platform for testing treatment modalities and could be monitored with ultrasound.

**Patients and methods:**

One million human hepatoma cells (Hep3B) were injected into the left lobe of the liver of immunocompromised mice. Tumor volume was monitored with high frequency-ultrasound until it reached 1,000mm^3^. At that time animals were sacrificed and examined for gross metastatic disease. Tumor sections were analyzed with hematoxylin and eosin (H&E) staining.

**Results:**

Tumor formed in 8/15 mice. The tumor was detected as small as 19.59mm^3 ^on ultrasound. Of the forming tumors, tumor size was 145±177.93mm^3^ at 60 days post-injection, 665±650.39mm^3 ^at 67 days, and reached >1000mm^3 ^by 76.6±9.9 days. At necropsy, four mice (50%) had tumor only within the liver, four (50%) had additional tumors in omentum, pelvis and peritoneum. H&E showed tumor within the normal liver parenchyma, with multiple mitotic figures, small areas of necrosis, and hemorrhage within the tumor.

**Conclusion:**

We have successfully established an orthotopic hepatoma murine model, with a local metastatic rate of 50%. Non-invasive tumor monitoring is feasible via ultrasound.

## Introduction

 In the pediatric population, liver tumors account for 1-4% of all tumors (1). The two most common types of liver tumors are hepatoblastoma, accounting for 43% of cases in infants and toddlers up to age 4, and hepatoma accounting for 23% in school aged and adolescent children (2). These tumors can be associated with hepatitis B and C, underlying cirrhosis, or inborn metabolic disorders (1, 3). However, many of these tumors arise without any underlying etiology. Due to the de novo formation and nonspecific symptoms, ranging from abdominal mass to vague abdominal pain, diagnosis usually is late and patients present with a greater disease burden (3). Patients who were diagnosed with hepatoma respond poorly to chemotherapies and had a low survival rate: 33% at 2 years, and 16.6% at 5 years (4), with reported rates of regional metastasis of 35% and distal metastasis of 34% (5). 

Curative treatment involves complete tumor resection. However, given proximity to vital structures and size of these tumors at presentation, this may not be feasible in many cases (3). Treatment can be multimodal with both resection and chemotherapy. Most commonly, a combination of cisplatin and doxorubicin is being used with improved outcomes (3). Neoadjuvant therapy has been shown to increase the likelihood of achieving a complete resection by 50%. However, systemic chemotherapies are not without serious side effects. Additionally, for metastatic hepatoma, chemotherapy has been investigated for use in palliation, but does not prolong life (6).

Murine models have been considered as an important testing platform to help with testing new treatment strategies in vivo, prior to human testing (7). There are many well-established orthotopic models for a variety of cancers, including neuroblastoma and hepatoma (7-10). An orthotopic tumor model takes advantage of the local tumor microenvironment and can mimic the clinical scenario more closely (11). We aimed to create an orthotopic hepatoma murine model using the Hep3B cell line and monitor the tumor growth with high frequency ultrasound as a platform for testing different treatment modalities. 

## Patients and Methods

**Cell Culture**

Hep3B (ATCC, Manassas, VA), a human hepatoma tumor cell line cultured from a pediatric patient, was used. Hep3B cells were cultured in media containing Dulbecco’s Modified Eagle’s Medium (DMEM), with 10% fetal bovine serum, and 100 IU ml^−1^ penicillin, and 100 *μ*g ml^−1^ streptomycin. The cell line was incubated at 5%CO_2 _atmosphere at 37°C and trypsinized and split at 90% confluence.

**Orthotopic Animal Model:**

Approval for all animal procedure was obtained from the Institutional Animal Care and Use Committee with guidance from the University of Illinois Chicago Animal facilities. All procedures were completed using athymic NCr nude female mice (Harlan, Indianapolis, IN) at 7 weeks of age. Procedures were completed under general anesthesia, using ketamine 100mg/kg and xylazine 19mg/kg.

At 7 weeks of age, a midline laparotomy incision was made on the mouse. The intestines were retracted inferiorly and laterally, exposing the left lobe of the liver. Briefly, 10^6 ^Hep3B cells suspended in 2μL of phosphate buffered saline, was measured using a Hamiton (Reno, NV) 5μL syringe and injected using a 30G needle into the sub-capsular compartment of the left liver. Direct pressure using a sterile cotton tip applicator was applied to the injection site to prevent cellular spillage. Upon completion, the abdomen was closed in two layers. Animals were monitored post operatively and return to housing after appropriate recovery from anesthesia.

**Tumor monitoring via high frequency** **ultrasound**

Animals underwent tumor growth and surveillance using the VisualSonics Vevo 2100 Sonographic probe. The mouse was secured to stage in the supine position. Using ultrasound guidance, the liver and gallbladder were identified, and screening for tumors was performed. When the tumor was identified, serial cross-sectional images (0.076 mm between images) were taken. The tumor volume was measured using the 3-D reconstruction tool (Vevo Software v1.6.0).

**Tumor Analysis**

When the tumor size reached>1000mm^3^, the animal was euthanized, and the chest and abdominal cavities were exposed to look for evidence of metastatic disease. The tumor and any metastatic tumor implants were removed and fixed in 10% buffered formalin. These tissues were then embedded in paraffin, and 5μm thick sections were cut and placed on glass slides. The tumor sections were stained with hematoxylin and eosin (H&E) and analysis was performed under the light microscopy.

**Statistical Analysis**

Statistical analysis was done using Student’s t-test where appropriate. A p-value of <0.05 was considered statistically significant. 

## Results

**Figure 1:**
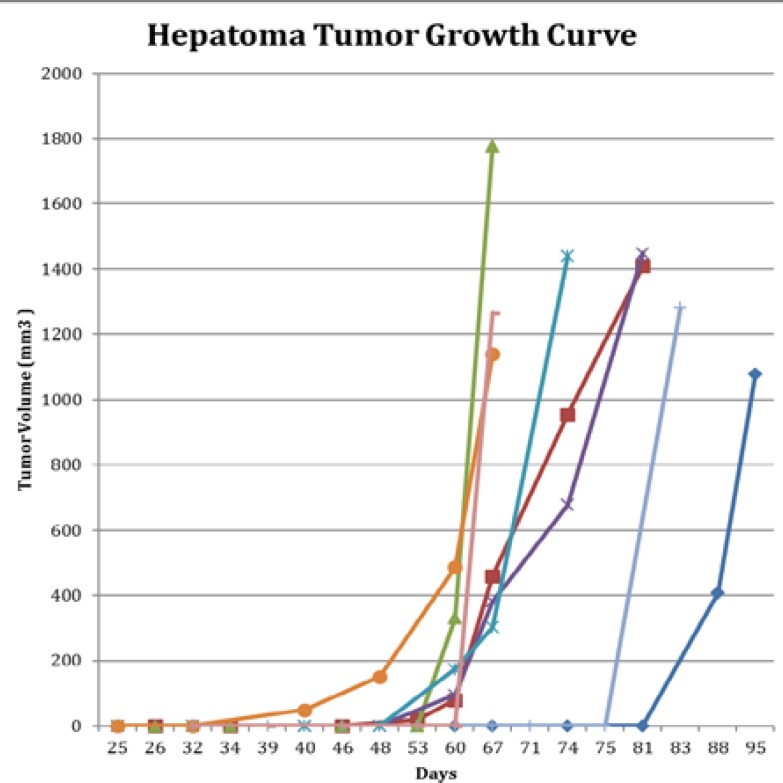
Growth curve of hepatoma tumor. Earliest tumor was identified on high frequency ultrasound at 40 days post injection

**Figure 2:**
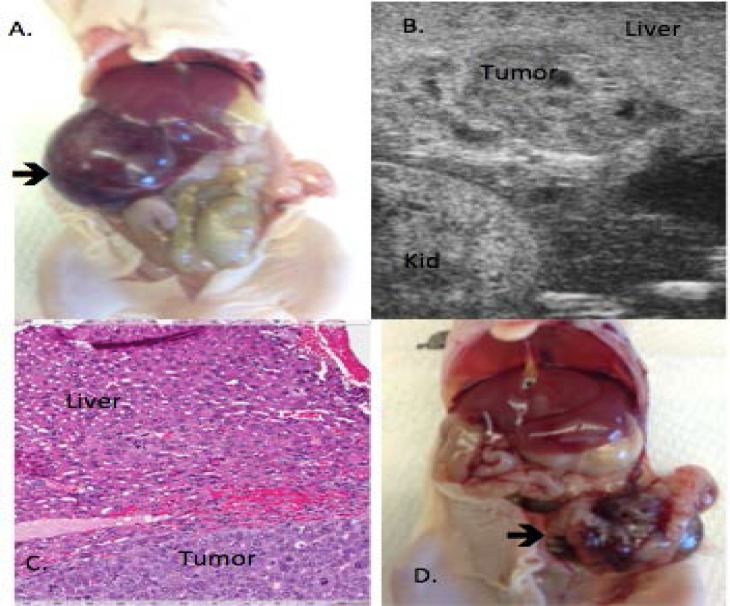
Hepatoma in Vivo Findings A. Orthotopic hepatoma tumor (arrow) in liver. B. Ultrasound image of hepatoma tumor, with surrounding normal liver parenchyma. Kid: kidney. C. H&E of tumor/liver interface. D. Metastatic tumor to pelvis.

Fifteen female nude mice were injected with 10^6^ Hep3B cells. Weekly sonographic surveillance was performed to scan the liver for tumor formation. When tumors formed within the liver, they were well circumscribed and heterogeneous, which were easily distinguished from the normal homogenous liver background (Figure 2B). The earliest tumor formation was detected at 40 days post injection, and animals were monitored up to 20 weeks post injection for tumor formation. A total of 8/15 mice formed tumors within this time period. The mean length of time to the initial development of the tumor was 63.88±15.50 days post injection. The smallest tumor size detected with ultrasound was 19.54mm^3^. Of the mice that developed tumors, tumor size was 145.75±166.44 mm^3 ^at 60 days post injection, 665.75±608.48mm^3^ at 67 days, and 767.745±520.46mm^3 ^at 74 days, respectively. On average, of the tumors identified between 80 to 200mm^3^, it took 19.25±3.03 days after detection to reach >1000mm^3^ (Figure 1).

Weekly weights were measured for all mice. When comparing those mice that failed to form tumors with those who formed tumors, there was no difference in the starting weight between the two groups, 17.99±1.29g versus 18.83±1.85g, respectively (p=0.48). At 60 days, non-tumor forming animals weighed 23.45±1.63g and those with tumors weighed 22.41±1.12g. No difference was observed between the two groups (p=0.25) (Table 1).

**Table 1:** Mean weight comparisons between tumor forming mice and non-tumor forming mice. There was no difference between the groups during the course of the experiment

Days post injection	Weight of animals without tumor (g)	Weight of animals with Tumor (g)	P-value
0	17.99±1.29	18.83±1.85	0.48
32	22.48±2.29	21.94±0.62	0.7
60	23.45±1.63	22.42±1.12	0.25

Once tumors reached >1000mm^3^, the animal was euthanized and underwent necropsy. At the time of necropsy, findings from ultrasound, including tumor size and position within the liver were compared to gross findings (Figure 2A, D). Animal necropsy confirmed the accuracy of ultrasound findings with the expected tumor volume in all mice of whom tumor was identified on ultrasound. Additionally, ultrasound and necropsy tumor findings were concordant, with no additional tumors identified at the time of necropsy that were not seen on ultrasound. No multifocal tumor was detected within the liver in neither the metastatic nor non-metastatic groups. Additionally, after mice reached 20-week post injection without evidence of tumor growth, no tumor was appreciated at the time of sacrifice. Of eight mice with tumors, intraperitoneal metastatic disease was seen in four mice. Areas of metastasis included pelvis, peritoneum, and omentum (Figure 2).

Tumors were sent for histologic evaluation with H&E staining. Well-circumscribed tumors were found with a background of the normal liver parenchyma, which demonstrated normal hepatocytes (Figure 2C). There were large pockets of tumor cells within the normal liver parenchyma. Multiple mitotic figures were identified within the tumor, with areas of hemorrhage and necrosis within the tumor. H&E staining of metastatic lesions revealed similar tumor architecture to the primary tumor: an intact capsule as well as evidence of hemorrhage however metastatic lesions had slightly more necrosis seen than the primary tumor. 

## Discussion

We found that an orthotopic murine model using a Hep3B cell line could be created and those local metastases were present in half of the animals with established primary liver tumor. The metastases were limited to the abdominal cavity. The high frequency ultrasound allowed longitudinal non-invasive monitoring of the tumor growth, and detected tumors before any significant mass can be detected grossly. Primary liver tumors as well as the local metastases had typical gross and histologic appearance of a hepatoma. Furthermore, the creation of an orthotopic tumor did not significantly change the animals’ weight. 

An orthotopic murine model using the Hep3B cell line has been previously described by Yao, et al. in 2003 (9). In that report, two million cells, compared to one million cells in our study, were injected into the left lobe of the liver, and cells were suspended in a higher volume of phosphate buffered saline 30-50μL, compared to 2μL in our study (9, 10). Tumors were established at a higher rate of 72.88% was described by Yao, et al., but interestingly tumor was only established within the liver, without other local metastasis noted (9). Smaller volume of injected cell suspension minimized the risk of spillage during implantation, assuring a localized tumor formation. Furthermore, this cell line has been described previously as a non-metastatic model (9, 10). Ma, et al. found that there were multiple nodules within the liver, but tumor never extended beyond the liver (10). Our model showed a rate of metastasis of 50% to peritoneum, pelvis and omentum, making our model unique. While metastatic hepatoma models have been established using Hep3B cell line via injection into the portal vein (12), spontaneous intraperitoneal metastasis has not been previously described. Our model offers a new platform to test innovative treatments for advanced metastatic hepatoma, which is associated with the greatest mortality (5). 

Orthotopic tumor models are integral in testing outcomes of experimental interventions due to their ability to simulate natural disease states (7, 9). However, one of the challenges associated with these models, is the ability to monitor tumor growth. In the subcutaneous models of hepatoma, calipers have been used for tumor monitoring (13). However, in the orthotopic models, caliper measurements are not feasible and accurate (14). Possible monitoring strategies for orthotopic hepatoma models include fluorescent protein monitoring or serial alpha feta protein monitoring (9, 15). However, these methods do not allow for direct measurement of tumor volume throughout the treatment course. 

We previously reported ultrasonography as an effective noninvasive monitoring technique for orthotopic neuroblastoma tumor monitoring with reasonable accuracy and reproducibility (8). Schmitz, et al. found that the growth of orthotopic hepatoma tumor, with cell line Hepa129, could be monitored with ultrasound. Wang, et al. reported their ability to detect hepatoma tumor development 86% of the time (16, 17). We found that we were able to detect tumor establishment and monitor hepatoma tumor growth 100% of the time, and tumor size was detected as small as 19.54 mm^3^.

Our model offers a mechanism to accurately monitor hepatoma growth in an orthotopic model with an intra-abdominal metastatic rate of 50%. A retrospective review of the SEER database of 218 patients showed that 69% of hepatoma patients were found to have regional, tumor extension into adjacent organs or regional lymph nodes, or distant tumor staging (5). The 5-year survival ranged between 10-26% (5). Czauderna, et al. identified metastatic disease as the most potent predictor of poor outcomes, occurring in 31% of their patients (18). Given the grim prognosis associated with metastatic disease, our murine model with both primary tumor and local metastasis can offer a unique platform for further investigation on treatment options.

 In conclusion, we have established an orthotopic hepatoma murine model, with a local metastatic rate of 50%. The gross appearance and histologic characteristics of the primary and metastatic tumors formed are consistent with clinically diagnosed hepatoma. Non-invasive longitudinal tumor detection and monitoring are feasible and accurate via high frequency-ultrasound. This model can be a valuable tool in testing novel treatment strategies.
